# Tensile Mechanical Properties and Dynamic Constitutive Model of Polyurea Elastomer under Different Strain Rates

**DOI:** 10.3390/polym14173579

**Published:** 2022-08-30

**Authors:** Yu Chen, Hui Guo, Minqian Sun, Xiao Lv

**Affiliations:** 1 School of Civil Engineering and Architecture, Southwest University of Science and Technology, Mianyang 621010, China; 2 State Key Laboratory of Disaster Prevention and Mitigation of Explosion and Impact, Army Engineering University of PLA, Nanjing 210007, China; 3 School of Geology Engineering and Geomatics, Chang’an University, Xi’an 710072, China

**Keywords:** polyurea elastomer, SHTB, mechanical properties, strain rate effect, dynamic constitutive model

## Abstract

In order to clearly explain the large deformation mechanical characteristics of polyurea under impact and to construct a dynamic model that can be used for finite element analysis, two kinds of polyurea materials were prepared by formula design, and their uniaxial tensile properties were tested with strain rates ranging from 10^−3^~10^3^ s^−1^ using an electronic universal testing machine and a split Hopkinson tensile bar (SHTB). The tensile stress–strain curves of polyurea were obtained under different strain rates. The difference in tensile mechanical properties of the materials was analyzed under dynamic loading and quasi-static loading. Based on the nonlinear viscoelastic theory and the energy dissipation rate inequality, a dynamic visco-hyperelastic constitutive model of polyurea elastomer was established. The research results showed that the uniaxial tensile stress–strain curves of two kinds of polyurea at different strain rates had obvious nonlinear characteristics and strain rate sensitivity and that their tensile strength increased with increased strain rate. The polyurea gradually changed from exhibiting rubbery mechanical behavior under quasi-static loading to glassy mechanical behavior under dynamic loading. The fitting analysis of experimental data and the results of finite element simulation showed that the dynamic constitutive model can predict the nonlinear mechanical behavior of polyurea elastomers over a wide range of strain rates. The research results could contribute to a deepening of the understanding of the damage and failure behavior of polyurea under impact load and provide a theoretical basis for numerical studies on impact safety design of polyurea-coated protective structures.

## 1. Introduction

Polyurea is a thermosetting elastomer polymer formed by isocyanate semi-prepolymer components and amine-terminated resin blend components through a chain-extension crosslinking reaction. It is low-cost and lightweight, and due to its unique microphase separation structure, it also has good wear resistance, impact resistance, corrosion resistance, flame retardancy and waterproof ability, so it is an ideal choice for many engineering applications [1]. Furthermore, polyurea elastomer can be easily coated and cures rapidly, with strong adhesion to substrates, including concrete and metal, which can ensure long-term coating without falling off. These technical advantages of polyurea elastomer contribute to its considerable application potential in military and civilian fields. To date, polyurea elastomer has been widely used for waterproofing, anticorrosion, explosion proofing and impact resistance [2,3]. In order to satisfy the use requirements of polyurea in these engineering applications, it is necessary to systematically study the mechanical behavior of polyurea under different loading rates. In addition, the establishment of a dynamic constitutive model of polyurea elastomer is helpful for designers to carry out finite element dynamic analysis of the protective structure of polyurea-based composites.

Since the US military first proposed the use of elastomer coating materials as a structural protective reinforced coating technology in the early 21st century, experimental and theoretical studies on the mechanical properties and deformation mechanisms of the elastomers under different loading conditions have been carried out successively [4,5]. Zaghloul et al. [6,7] studied the fatigue and tensile mechanical properties of fiber-reinforced thermosetting polyester elastomer materials at different strain rates and concluded that reasonable material design and appropriate fiber volume fraction can significantly enhance the mechanical properties of such materials. Sarva and Shim et al. [8,9] carried out uniaxial compression tests on polyurea elastomer under different strain rates and found that polyurea had the correlation characteristics of high hyperelastic deformation and strain rate according to an analysis of its stress–strain relationship. Pathak et al. [10] analyzed the uniaxial tensile mechanical properties of polyurea elastomer at strain rates of 0.15~830 s^−1^ and found that with increased strain rate, its strength increased, and the failure strain decreased. Roland et al. [11] studied the uniaxial tensile properties of polyurea in the strain rate range of 0.06~573 s^−1^ using a drop-weight tester and found that its tensile strength increased with increased strain rate. 

Some researchers have also proposed parameterized constitutive models of polyurea elastomer, such as the Ogden model, the Arruda-Boyce model, the Yeoh model, the Mooney–Rivlin model, etc. [12,13,14,15], although the strain rate effect was not taken into account in these models. In terms of constitutive modeling of polyurea, Amirkhizi et al. [16] proposed a linear viscoelastic constitutive model including viscous effect based on the WLF time–temperature conversion equation, which describes the stress–strain behavior of polyurea at low strain rates. Subsequently, Elsayed and Gong et al. [17,18] proposed more complex constitutive models, which can better describe the experimental results of polyurea under uniaxial loading, but their fitting effect was not ideal under complex stress conditions, and many parameters in these models presented with considerable discrepancies. Therefore, in this paper, we intend to develop a dynamic constitutive model based on the tensile experimental data of polyurea elastomer that can fit the stress–strain curves of the material under any stress state using a set of material parameters and obtain stable results under different loading conditions to calculate the selected stress tensor expression in any coordinate system.

In the present study, the quasi-static and dynamic tensile mechanical properties of two polyurea elastomers were tested using an electronic universal testing machine and a low-impedance SHTB device. The uniaxial tensile stress–strain curves of the two materials in the strain rate range of 10^−3^~10^3^ s^−1^ were obtained, and the variation law of tensile mechanical properties of polyurea elastomers under loading from low strain rates to high strain rates was analyzed. Furthermore, a dynamic constitutive model describing the nonlinear mechanical behavior of polyurea was constructed based on the experimental data and theoretical derivation. The constitutive model was applied to the finite element simulation, and its validity was verified.

## 2. Experiments

### 2.1. Experimental Material

The two polyurea materials used in this study were both prepared by spraying two components, A and B, through high-temperature and high-pressure collision technology to make films, in which the stoichiometric ratio of A and B were determined according to an isocyanate index of 1.05. The isocyanate index represents the excess degree of isocyanate in the polyurea formula, that is, the ratio of the equivalent amount of -NCO in component A to the equivalent amount of -OH in component B. Theoretically, -NCO needs to react with -OH, but it is often unable to reach the ideal state in actual production because -NCO easily reacts with active hydrogen in atmospheric water during storage, resulting in a reduction in -NCO content. Therefore, the isocyanate index in the polyurea formulation was 1.05 in this experiment.

Component A is the isocyanate semi-prepolymer formed by the reaction of isocyanate MDI-50 and polyether polyol PTMG 1000, in which the isocyanate content (-NCO%) was 15.5%. The preparation process of component A was as follows: first, polyether polyol was added to a reaction kettle and dehydrated under reduced pressure for 1.5~2 h at a temperature of 95~120 °C until the water content of the polyether polyol was ≤ 0.05%; then, the temperature was lowered to 60 °C, isocyanate was added, the temperature was raised to 80 °C and the reaction was maintained for 2.5~3 h. After the reaction was completed, the temperature was lowered to below 60 °C, and the material was discharged to detect the content of -NCO in the semi prepolymer, which was stored in a closed container filled with nitrogen after reaching the control index.

Component B was a mixture of amino terminated polyether, amine chain extender and additives, with chain extenders E100 and W6200 and amino-terminated polyethers D2000 and T5000. After repeated debugging, the ratio of amine chain extenders E100 to W6200 in the two polyurea materials was 5:2, and the ratio of amino-terminated polyethers D2000 to T5000 was 12:1 and 6:1, which were called PU-1 and PU-2 polyurea, respectively. The preparation method of component B was as follows: the liquid amine chain extenders, amino-terminated polyethers and functional additives were added to a closed reaction kettle in sequence, evenly stirred for 1~2 h and dehydrated under reduced pressure for 1.5~2 h at 90~100 °C; then, the obtained component B was filtered and encapsulated, and the water content was determined by Karl Fischer method to be ≤0.05%.

In this study, a two-component electric spraying machine was used to spray components A and B on polytetrafluoroethylene plates after being evenly mixed at high temperature and high pressure. During spraying, the temperature of the spraying machine was set at 65 °C, and the flow rate was set at 2300 kg/min. The thickness of two kinds of polyurea coating under dry conditions was 2 mm. After coating, they were cured at room temperature for 7 days to demold and were then cut out as samples with a punching machine for performance testing.

**Figure 1:**
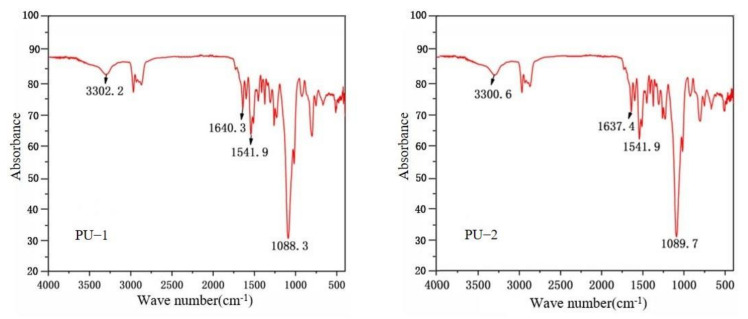
Fourier transform infrared absorption spectra of polyurea samples.

The physicochemical properties and parts by mass of the main raw materials of the two synthesized polyurea are shown in Table 1. The molecular structure of the synthesized PU-1 and PU-2 were characterized by a Fourier transform infrared spectrometer, and the infrared spectra are shown in Figure 1. The stretching vibration peaks of N-H groups in the molecular structures of PU-1 and PU-2 appear at wave numbers 3302.2 cm^−1^ and 3300.6 cm^−1^, the stretching vibration peaks of hydrogen-bonded C=O groups appear at wave numbers 1640.3 cm^−1^ and 1637.4 cm^−1^, the stretching vibration peaks of C-N groups appear at wave numbers 1541.9 cm^−1^, and the stretching vibration peaks of C-O-C groups appear at wave numbers 1088.3 cm^−1^ and 1089.7 cm^−1^, respectively. Comparative analysis of these stretching vibration peaks and the characteristic vibration peaks of polyurea reported in the literature [19] proves that the synthesized samples have the molecular structural characteristics of polyurea.

### 2.2. Experimental Technology

**Figure 2:**
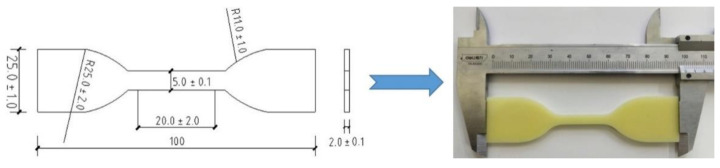
Size and schematic drawing of the polyurea sample for the quasi-static test.

In this paper, a quasi-static tensile test of polyurea elastomer was carried out using an electronic universal testing machine with a measuring range of 20 kN. The test adopted the displacement control mode, with an adjustment range of displacement rates of 0.005~500 mm/min and a control accuracy of less than ±1%. The tensile load and gauge segment displacement of the polyurea samples were recorded by the load and displacement sensors of the electronic universal testing machine, and the tensile stress–strain curve of polyurea elastomer at low strain rates was obtained by calculation of the combined sample size. According to standard GB/T528-2009 [20], the polyurea sample for quasi-static test was designed as shown in Figure 2. During the experiment, the tensile test at each strain rate was repeated three times, and the average value of three experimental results was taken to ensure its effectiveness.

**Figure 3:**
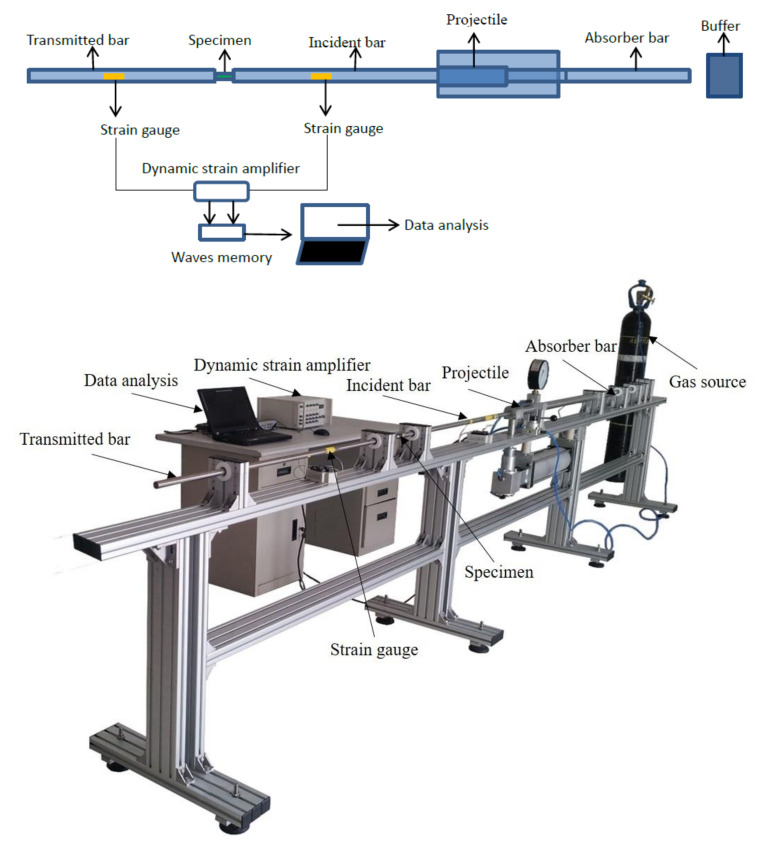
Schematic diagram and photos of the SHTB device used in this study.

**Figure 4:**
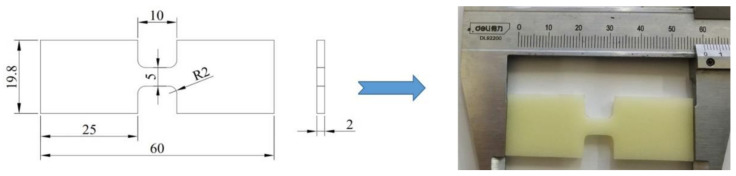
Size and schematic drawing of the polyurea sample for dynamic testing.

**Figure 5:**
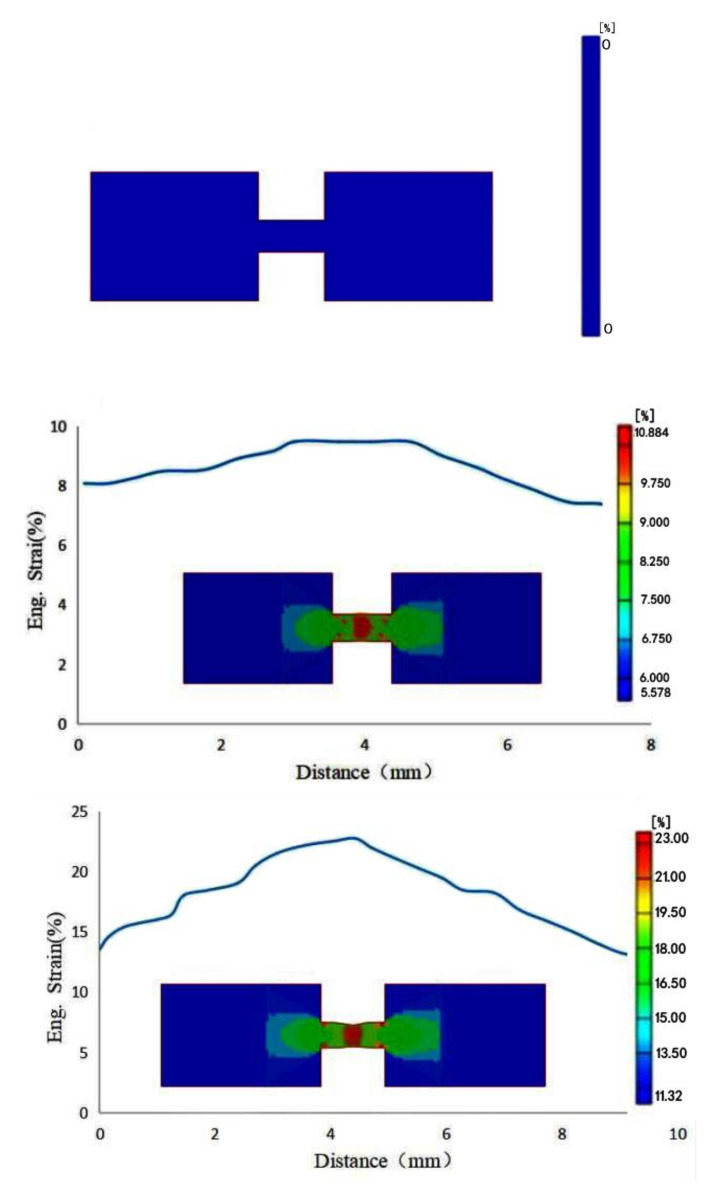

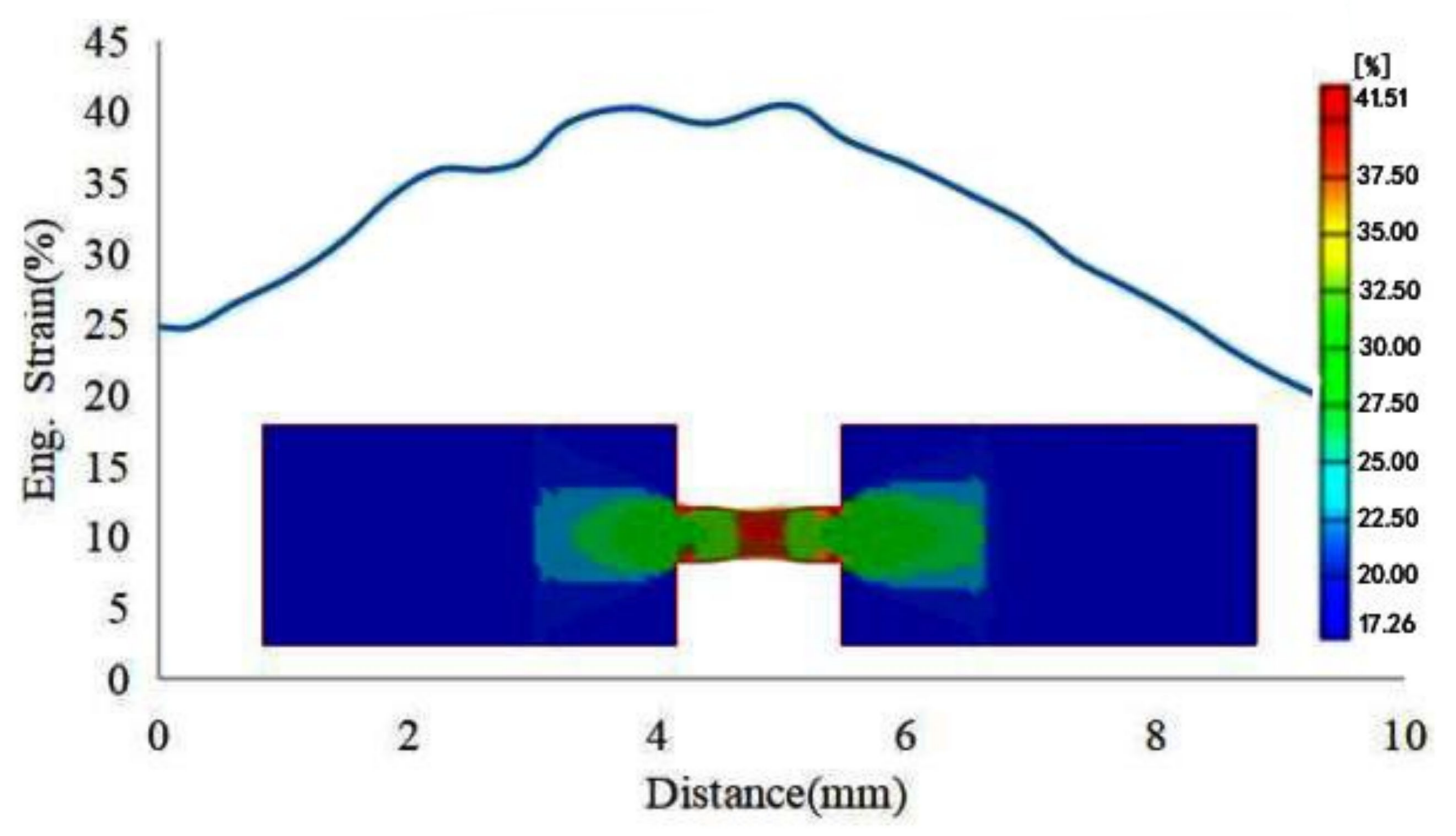
Quantitative strain field of the polyurea sample obtained by numerical simulation.

A dynamic tensile test of polyurea elastomer was carried out on a low-impedance SHTB device, which was mainly composed of a transmitting cylinder, an impact bar, an incident bar, a transmitted bar and a data acquisition system. During the test, the impact bar driven by high-pressure gas struck the flange at the end of the incident bar to form a tensile stress wave, and then the sample was loaded. The tensile stress amplitude was measured and transmitted to the data processing system by the strain gauge attached to the incident bar and transmission bar for storage and processing; then, the dynamic tensile stress–strain curve of the polyurea sample was obtained according to one-dimensional stress wave theory. A schematic diagram and photos of the SHTB device used in this study are shown in Figure 3. The incident bar and transmission bar were both aluminum with low wave impedance, with a diameter of 20 mm. Due to the low wave impedance of polyurea, in order to avoid the problem of weak transmission signal caused by the impedance mismatch between the sample and the bars during the test, aluminum bars with low wave impedance were used, and the amplitude of the transmission signal was improved by controlling the bridge voltage and gain. Before the dynamic test, a circular copper sheet was attached to the center of the incident bar as a pulse shaper, which was conducive to improving the stress uniformity of the sample and suppressed the high-frequency components in the stress pulse, reducing the influence of the dispersion effect on the experimental results. The size and shape of the polyurea dynamic tensile sample shown in Figure 4 were designed according to the ASM manual and research on the sample size of soft material for dynamic testing reported in the literatures [21,22]. During the test, the polyurea sample was connected to the fixture by epoxy adhesive method, and the fixture was connected with the ends of incident bar and transmission bar through threaded holes. In order to verify the stress uniformity of the polyurea sample under dynamic tensile loading, a deformation process was simulated by Ls-Dyna finite element software to obtain the quantitative strain field, as shown in Figure 5. The results showed that the engineering strain in the gauge section of the sample was close to the calculated value. The strain field in the gauge segment of the sample was relatively uniform in the middle and later stages of deformation. The strain near the transmission bar (left end) of the sample was slightly less than that near the incident bar end. However, after the engineering strain in the gauge section of the sample exceeded 3%, the strains at both ends were basically equal, which can be approximately regarded as uniform deformation of the sample.

## 3. Analysis of Experimental Results

**Figure 6:**
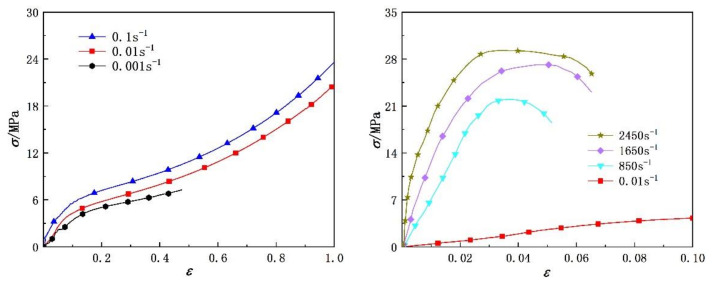
Quasi-static and dynamic tensile stress–strain curves of PU-1 polyurea elastomer.

**Figure 7:**
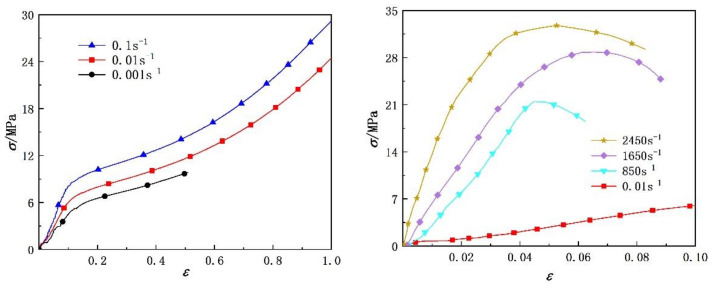
Quasi-static and dynamic tensile stress–strain curves of PU-2 polyurea elastomer.

Based on the experimental method described in the previous section, the tensile stress–strain curves of the two polyurea elastomers at different strain rates are shown in Figure 6 and Figure 7, respectively. Both polyurea materials exhibited characteristics of rubber-like hyperelastic large deformation under quasi-static loading, and the deformation process can be roughly divided into two stages: linear elasticity and high elasticity. In the linear elasticity stage, the strain range was about 0~0.1, the stress–strain relationship approximately conformed to Hooke’s law and the true stress increased linearly with the true strain. During this stage, the true stress value of polyurea was low, and the soft segments in its molecular chain had not overcome the inhibition of hard segments, so its deformation was small. As the true stress value of polyurea continued to increase, its strain increased sharply and reached the high elasticity stage of the stress–strain curve. During this stage, the soft segments of the polyurea molecular chain began to overcome the binding of hard segments and deform. Because the glass transition temperature of the polyurea soft segment was lower than room temperature, it exhibited characteristics of low modulus and large deformation. The deformation of the two polyurea materials under dynamic loading was small, and the strain value did not exceed 0.1, showing the elastic brittle deformation characteristics of glass-like materials. The deformation process can be roughly divided into two stages: linear elastic and nonlinear elastic hardening. In conclusion, polyurea elastomers gradually changed from exhibiting rubbery mechanical characteristics at low strain rates to glassy mechanical characteristics at high strain rates. This may be due to the fact that at high strain rates, the soft segments of the polyurea molecular chain did not have enough time to overcome the limitation of the hard segments, and only small motion units, such as bond length, bond angle and side groups, moved. When polyurea was subjected to short-term impact, it could only adapt to the external force by changing the bond length and bond angle on the main chain, resulting in a much lower deformation capacity under dynamic loading than that under quasi-static loading.

Figure 6 and Figure 7 show that the uniaxial tensile stress–strain curve of polyurea under different strain rates exhibited significant strain rate sensitivity, that is, with increased strain rate, higher stress values were required to achieve the same deformation. In addition, comparison of the stress–strain curves of the two polyurea elastomers shows that the tensile strength of the PU-1 polyurea was slightly lower than that of PU-2, mainly due to a functionality of amino polyether D2000 in the original component of polyurea of 2, whereas the functionality of T5000 was 3. The amount of T5000 in the original component of PU-2 polyurea was greater than that of PU-1, so it was more prone to a crosslinking reaction and to form more reticular molecular chains.

## 4. Dynamic Constitutive Model

In order to describe the mechanical characteristics of polyurea elastomer under dynamic loading, the expression of partial stress tensor was derived based on the energy dissipation rate inequality derived from the law of energy conservation and the law of entropy increase in the statistical thermodynamic theory. By substituting the classical hyperelastic strain energy density function and viscoelastic strain energy density function into the expression of partial stress tensor, the dynamic constitutive model of polyurea elastomer can be derived by superimposing hydrostatic pressure and loading boundary conditions. In continuum mechanics, the local mapping between the initial configuration (*X*) and the current configuration (*x*) of a material in the deformation process can be described by the deformation gradient tensor (***F***), which can be expressed as [23]:

(1) $$F=\partial x/\partial X=\sum_{i=1}^{3}\lambda_{i}k_{i}⊗K_{i}$$

where *X* is the coordinate of the typical material point in the reference configuration, *x* is the coordinate of the typical material point in the current configuration, *λ*_i_ is the principal elongation and *λ_i_* = 1 + *ε_i_*, k*_i_* and K*_i_* are coordinate unit vectors. Thus, the left Cauchy–Green tensor (B) describing the material under finite deformation is:

(2) $$B=FF^{T}=\sum_{i=1}^{3}\lambda_{{}_{i}}^{2}k_{i}⊗k_{i}$$

The three principal invariants of the left Cauchy–Green tensor (***B***) can be obtained by the following formula:

(3) $$\left\{\begin{array}{l} I_{1}=\mathrm{tr}B\\ I_{2}=\frac{1}{2}\left[{\left(\mathrm{tr}B\right)}^{2}-\mathrm{tr}B^{2}\right]\\ I_{3}=\det B=J^{2} \end{array}\right.$$

where *J* is the Jacobian determinant of the deformation gradient. For polyurea elastomers, the volume is approximately incompressible, so ***I***_3_ = 1. The deformation rate tensor (***D***) in the current configuration can be calculated by the following formula:

(4) $$D=\frac{1}{2}\left[\dot{F}F^{-1}+{\left(\dot{F}F^{-1}\right)}^{T}\right]$$

Based on the law of energy conservation and the law of entropy increase in the statistical thermodynamic theory, the energy dissipation rate inequality of per-unit volume is given as follows [24,25]

(5) $$J\sigma_{\mathrm{ds}}:D-\dot{\zeta }\geq 0$$

where ***σ***_ds_ is the Cauchy partial stress tensor, and ζ is the Helmholtz free energy per unit volume, which is the elastic strain energy for an elastomer at constant temperature. The elastic strain energy per unit volume can be expressed as a function of three principal invariants as:

(6) $$\dot{\zeta }=\dot{\zeta }\left(I_{1},I_{2},\;I_{3}\right)=\dot{\zeta }\left(I_{1},I_{2},\;1\right)=\frac{\partial \zeta }{\partial I_{1}}{\dot{I}}_{1}+\frac{\partial \zeta }{\partial I_{2}}{\dot{I}}_{2}=\left[\frac{\partial \zeta }{\partial I_{1}}I+\frac{\partial \zeta }{\partial I_{2}}\left(I_{1}-B\right)\right]\cdot \dot{B}$$

Substituting Equation (6) into Equation (5) results in:

(7) $$\sigma_{\mathrm{ds}}:D-\left[\frac{\partial \zeta }{\partial I_{1}}I+\frac{\partial \zeta }{\partial I_{2}}\left(I_{1}-B\right)\right]\cdot \dot{B}=\sigma_{\mathrm{ds}}:D-\left[\frac{\partial \zeta }{\partial I_{1}}I+\frac{\partial \zeta }{\partial I_{2}}\left(I_{1}-B\right)\right]\cdot \left(2B:D\right)\geq 0$$

According to the entropy increasing theorem, the greater-than sign in the equation holds, indicating that the deformation process is irreversible, and the equal sign holds, indicating that the deformation process is reversible. For polyurea elastomers, the large hyperelastic deformation is reversible under external loading. Therefore, in order to ensure that Equation (7) is valid for any deformation process of the material, the coefficient of deformation rate tensor (***D***) should be zero so that the following constitutive relationship can be obtained:

(8) $$\sigma_{\mathrm{ds}}=2\left[\frac{\partial \zeta }{\partial I_{1}}B+\frac{\partial \zeta }{\partial I_{2}}\left(I_{1}B-B^{2}\right)\right]=2\sum_{i=1}^{3}\frac{\partial \zeta }{\partial \lambda_{i}^{2}}\left(FK_{i}\right)⊗\left(FK_{i}\right)=2\sum_{i=1}^{3}\lambda_{i}\frac{\partial \zeta }{\partial \lambda_{i}}k_{i}⊗k_{i}$$

In Equation (8), only the deformation effect of the materials is considered, and the effect of hydrostatic pressure (P) needs to be superimposed, which is obtained from the loading boundary conditions, that is:

(9) $$\sigma =-PI+\sigma_{\mathrm{ds}}=-PI+2\left[\frac{\partial \zeta }{\partial I_{1}}B+\frac{\partial \zeta }{\partial I_{2}}\left(I_{1}B-B^{2}\right)\right]$$

Due to the viscoelastic properties of polyurea elastomers, the material has not only superelastic deformation energy but also viscoelastic dissipation energy caused by intramolecular friction. Based on the theory proposed by Simo et al. [26,27], the time-dependent strain energy density function under finite deformation can be expressed as:

(10) $$\zeta =\zeta_{h}\left(E\right)+\zeta_{v}\left(E,t\right)$$

where ***E*** is the Green’s strain tensor, ζ*_h_*(***E***) is the hyperelastic strain energy density function and ζ*_v_*(***E***, t) is the viscoelastic strain energy density function related to the material deformation process. Some classical hyperelastic strain energy density functions have been reported in the literature, among which the Ogden strain energy density function is simple in form with suitable application in engineering [12]. Therefore, the second-order Ogden strain energy density function was adopted in the present study, which is expressed as follows:

(11) $$\zeta_{h}\left(E\right)=\sum_{j=1}^{2}\frac{2\mu_{j}}{\alpha_{j}^{2}}\left(\lambda_{1}^{\alpha_{j}}+\lambda_{2}^{\alpha_{j}}+\lambda_{3}^{\alpha_{j}}-3\right)$$

where *α_j_* and *μ_j_* are the undetermined parameters of the material. The strain energy density function of viscoelastic deformation was discussed in the research of Yang and Guo et al., which can better fit and compare the experimental results under different stress states with a group of material parameters [28,29]. Based on these studies, the viscoelastic strain energy density function can be expressed as follows:

(12) $$\zeta_{v}\left(E,t\right)=\int_{0}^{t}\frac{1}{\theta }e^{-\left(t-\tau \right)/\theta }\left[A_{1}\left(I_{1}-3\right)+A_{2}\left(I_{2}-3\right)+A_{3}\left(I_{1}-3\right)\left(I_{2}-3\right)\right]\dot{E}\left(\tau \right)d\tau$$

where *A*_1_, *A*_2_ and *A*_3_ are undetermined parameters, and θ is the relaxation factor. By superimposing the two strain energy density functions into Equation(9), the dynamic visco-hyperelastic constitutive relation of polyurea elastomer can be obtained as follows:

(13) $$\begin{array}{l} \sigma =-PI+\sum_{i=1}^{3}\left(\frac{4\mu_{1}}{\alpha_{1}}\lambda_{i}^{\alpha_{1}}+\frac{4\mu_{2}}{\alpha_{2}}\lambda_{i}^{\alpha_{2}}\right)k_{i}⊗k_{i}\\ \;+\int_{0}^{t}\frac{1}{\theta }e^{-\left(t-\tau \right)/\theta }\left[\left(2A_{1}+A_{3}\sum_{\begin{array}{l} i,k=1\\ i\neq k \end{array}}^{3}\lambda_{i}^{2}\lambda_{k}^{2}-6A_{3}\right)B+\left(2A_{2}+2A_{3}\sum_{i=1}^{3}\lambda_{i}^{2}-6A_{3}\right)\left(\sum_{i=1}^{3}\lambda_{i}^{2}\cdot B-B^{2}\right)\right]{\dot{\lambda }}_{i}d\tau \end{array}$$

The three-principal elongation of polyurea elastomer under uniaxial tension can be expressed as *λ*_1_ = *λ*, *λ*_2_ = *λ*_3_ = *λ*^−1/2^ and considering the boundary conditions of uniaxial tension and compression loading, *σ*_22_ = *σ*_33_ = 0, so Equation (13) can be simplified as:

(14) $$\begin{array}{l} s_{11}=\frac{4\mu_{1}}{\alpha_{1}}\left(\lambda^{\alpha_{1}}-\lambda^{-\alpha_{1}/2}\right)+\frac{4\mu_{2}}{\alpha_{2}}\left(\lambda^{\alpha_{2}}-\lambda^{-\alpha_{2}/2}\right)\\ \;+\frac{2}{\theta }\int_{0}^{t}\left[A_{1}\lambda^{2}+2A_{2}\lambda +A_{3}\left(4\lambda^{3}-3\lambda^{2}-6\lambda +5\right)\right]\exp\left(-\frac{t-t}{\theta }\right)\dot{\lambda }d\tau \\ \;+\frac{1}{\theta }\int_{0}^{t}\lambda^{-1/2}\left[A_{1}\lambda^{-2}+A_{2}\left(1+\lambda^{-3}\right)+A_{3}\left(\lambda^{2}-3+5\lambda^{-1}-3\lambda^{-2}-3\lambda^{-3}+3\lambda^{-4}\right)\right]\exp\left(-\frac{t-t}{\theta }\right)\dot{\lambda }d\tau \end{array}$$

**Figure 8:**
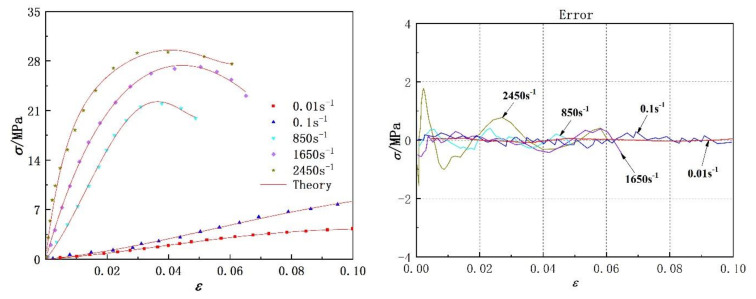
Comparison between model fitting results and experimental data of PU−1 polyurea and model prediction error.

**Figure 9:**
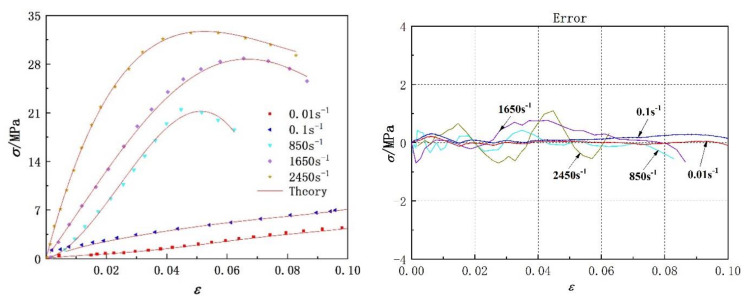
Comparison between theoretical results of the model and experimental data of PU−2 polyurea, as well as model prediction error.

**Figure 10:**
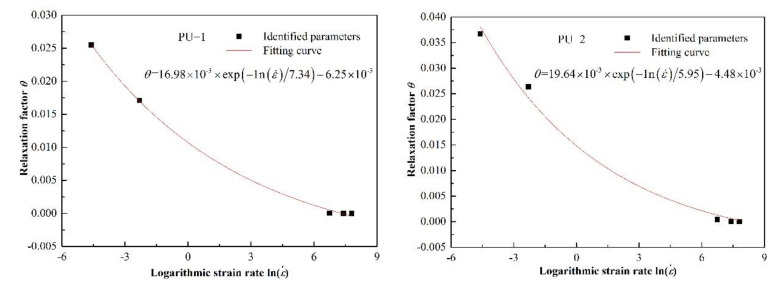
Variation in relaxation factor (θ) relative to the logarithmic strain rate.

The experimental data of two kinds of polyurea at different strain rates were fitted with Equation (14). Comparisons between the fitting results and the experimental data are shown in Figure 8 and Figure 9, respectively. The model parameters and fitting similarity (R^2^) obtained by fitting data with the least square method are shown in Table 2 and Table 3. The fitting parameters for the data of each polyurea were constant, except the relaxation factor (θ), mainly because it was related to the loading time. The variation in the relaxation factor (θ) relative to the logarithmic strain rate is shown in Figure 10. Numerical fitting showed that the relaxation factor (θ) has an exponential relationship with the logarithmic strain rate. Therefore, when using the newly proposed constitutive model to predict the dynamic mechanical behavior of polyurea elastomers, the exponential relationship shown in Figure 10 can be used to determine the value of the relaxation factor (θ) at different strain rates.

Figure 8 and Figure 9 show that the theoretical results were in agreement with the experimental data. The fitting effect of the model is remarkable at low strain rates and decreases slightly with increased strain rates. The residual stress error is approximately in the range of −2 MPa < Δ < 2 MPa and is negligible compared with the corresponding engineering stress amplitude. In addition, the parameters of the dynamic constitutive model were relatively stable, indicating that the proposed dynamic constitutive model is suitable for describing the nonlinear mechanical behavior of polyurea over a wide range of strain rates.

## 5. Verification of the Model by Finite Element Simulation

**Figure 11:**
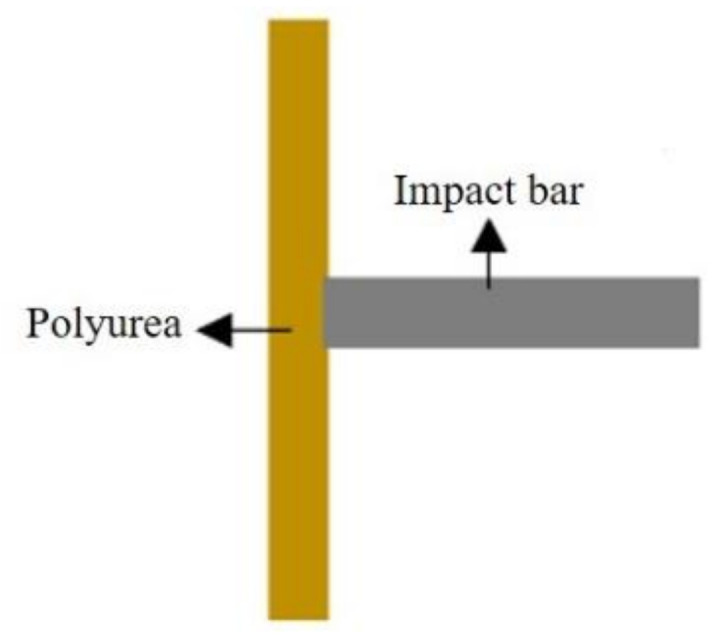
Numerical modeling by finite element software.

**Figure 12:**
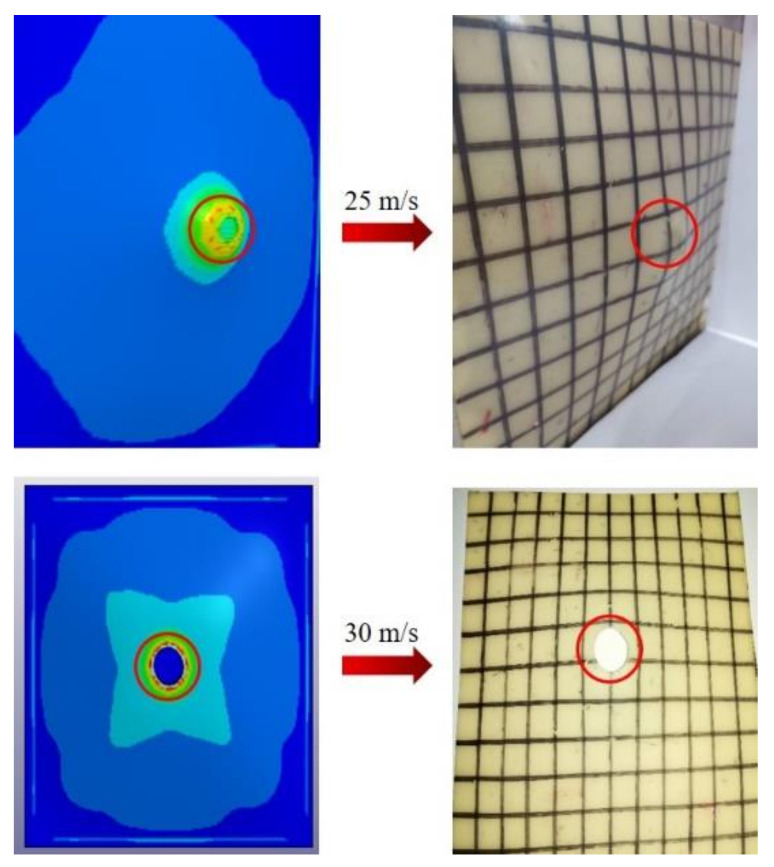
Comparison between numerical simulation and experimental results of the failure mode of polyurea at different impact velocities.

**Figure 13:**
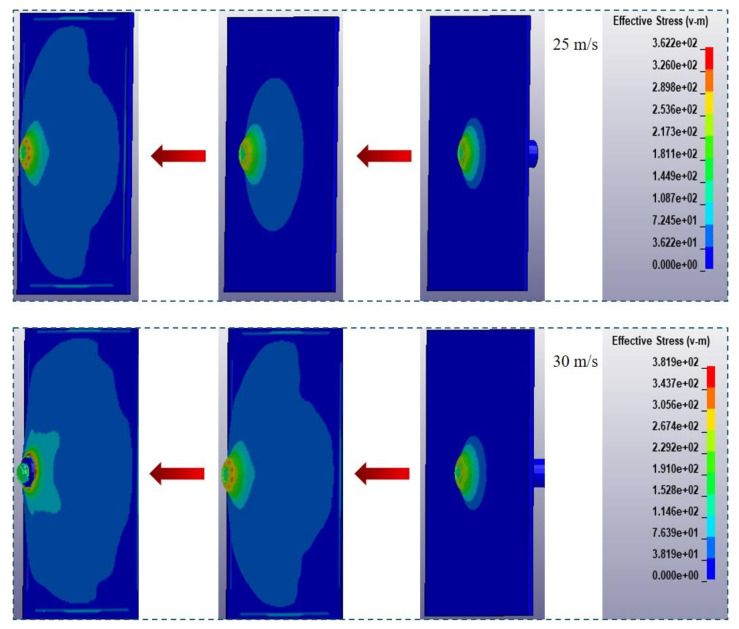
Typical stress cloud diagram of polyurea during the impact process.

A direct impact test of the polyurea coating material was carried out by using a split Hopkinson pressure bar device, to determine its deformation process and failure mode. The constitutive model established in this study was embedded into LS-DYNA finite element software through subroutine to simulate a direct impact test of the polyurea elastomer and compare the test results under specific conditions. In the direct impact test, a square PU-2 polyurea specimen with a side length of 200 mm and a thickness of 2 mm was used. The diameter of the impact bar was 20 mm, and the length was 200 mm. When using LS-DYNA finite element software for numerical simulation, full-scale modeling was used, as shown in Figure 11. The impact bar and polyurea were in surface-to-surface contact, the polyurea specimen was fixed and restrained on four sides and the impact bar was loaded by velocity. The material of the impact bar was LY12CZ aluminum alloy, and the material properties in the simulation used the built-in MAT-PLASTIC-KINEMATIC card in the finite element software. A comparison of failure modes of polyurea under impact load obtained by numerical simulation and testing is shown in Figure 12. The numerical simulation results are consistent with the experimental results, which confirms the effectiveness of using this model to characterize the properties of polyurea. The failure process of polyurea under impact load can be observed through finite element simulation. A typical stress cloud diagram of polyurea in the impact process is shown in Figure 13. The simulated equivalent stress of polyurea at failure is basically consistent with the failure stress obtained in the dynamic tensile test. After the impact bar comes into contact with the polyurea, the polyurea exhibits large tensile deformation. With increased impact speed, perforation failure of the polyurea ultimately occurs.

## 6. Conclusions

In the present study, the uniaxial tensile mechanical properties of two polyurea elastomers at different strain rates were investigated through quasi-static and dynamic mechanical tests. We found that polyurea exhibited rubbery mechanical characteristics of large deformation at low strain rates, and its deformation process can be divided into two stages: linear elasticity and high elasticity. However, polyurea exhibited glassy mechanical characteristics at high strain rates, and its deformation process can be divided into linear elastic and nonlinear elastic hardening. With increased strain rate, the uniaxial tensile stress–strain curve of polyurea showed obvious strain rate sensitivity, and higher tensile stress values were required to achieve the same deformation. The tensile strength of the PU-1 polyurea studied in this paper was slightly lower than that of PU-2, which mainly due to the large amount of T5000 in the original component of PU-2, which was prone to crosslinking reaction and formed more reticular molecular chains.

Based on the experimental data and mathematical derivation method, a dynamic visco-hyperelastic constitutive model describing the tensile mechanical properties of polyurea elastomer at different strain rates was constructed. Through comparison, we found that the theoretical curves generated by the model were in agreement with the experimentally obtained stress–strain curves. The constitutive model established in this paper was embedded into LS-DYNA finite element software to simulate the deformation and failure mode of polyurea elastomer under impact load. It was further confirmed that the proposed dynamic constitutive model can accurately predict the mechanical behavior of polyurea elastomer.

## Figures and Tables

**Table 1:** Physicochemical properties and parts by mass of raw materials for synthesized polyurea.

Component	Material	Molecular Weight	Relative Density	Viscosity (mPa.s)	Parts by Mass (PU-1)	Parts by Mass (PU-2)
A	MDI-50	250.25	1.22	15	53.35	53.35
	PTMG1000	1000	0.975	310	51.65	51.65
B	D2000	2000	0.991	248	60	54
	T5000	5000	0.997	819	5	9
	E100	178.3	1.02	280	25	26.4
	6200	310.48	0.99	115	10	10.6

**Table 2:** Model fitting parameters for experimental data of PU-1 polyurea.

Strain Rate/s^−1^	*α*_1_/MPa	*μ*_1_/MPa	*α*_2_/MPa	*μ*_2_/MPa	*A*_1_/MPa	*A*_2_/MPa	*A*_3_/MPa	θ	Fitting Similarity R^2^
0.01	0.003	13.8	−41.4	125.6	0.08	−0.039	−0.267	25.5 × 10^−3^	0.99875
0.1								17.1 × 10^−3^	0.99896
850								29.7 × 10^−6^	0.99762
1650								8.76 × 10^−6^	0.99463
2450								1.11 × 10^−6^	0.99581

**Table 3:** Model fitting parameters for experimental data of PU-2 polyurea.

Strain Rate/s^−1^	*α*_1_/MPa	*μ*_1_/MPa	*α*_2_/MPa	*μ*_2_/MPa	*A*_1_/MPa	*A*_2_/MPa	*A*_3_/MPa	θ	Fitting Similarity R^2^
0.01	0.003	14.3	−42.6	128.4	0.34	−0.17	−0.41	36.7 × 10^−3^	0.99952
0.1								26.4 × 10^−3^	0.99816
850								46 × 10^−5^	0.99543
1650								5.7 × 10^−5^	0.99872
2450								3.3 × 10^−5^	0.99793

## Data Availability

The data presented in this study are available on request from the corresponding author.
